# Geospatial epidemiology of leprosy in northwest Bangladesh: a 20-year retrospective observational study

**DOI:** 10.1186/s40249-021-00817-4

**Published:** 2021-03-22

**Authors:** Caroline A. Bulstra, David J. Blok, Khorshed Alam, C. Ruth Butlin, Johan Chandra Roy, Bob Bowers, Peter Nicholls, Sake J. de Vlas, Jan Hendrik Richardus

**Affiliations:** 1 Department of Public Health, Erasmus MC, University Medical Center Rotterdam, Rotterdam, The Netherlands; 2 Heidelberg Institute of Global Health, Heidelberg University Hospital, Heidelberg, Germany; 3 Rural Health Programme, The Leprosy Mission International Bangladesh, Nilphamari, Bangladesh; 4 The Leprosy Mission England and Wales, Goldhay Way, Orton Goldhay, Peterborough, England; 5 Menzies Health Institute Queensland, Griffith University, Brisbane, Australia; 6 Southampton, UK

**Keywords:** Leprosy, Epidemiology, Geospatial, Hotspots, Neglected tropical diseases, Patient characteristics

## Abstract

**Background:**

Leprosy is known to be unevenly distributed between and within countries. High risk areas or ‘hotspots’ are potential targets for preventive interventions, but the underlying epidemiologic mechanisms that enable hotspots to emerge, are not yet fully understood. In this study, we identified and characterized leprosy hotspots in Bangladesh, a country with one of the highest leprosy endemicity levels globally.

**Methods:**

We used data from four high-endemic districts in northwest Bangladesh including 20 623 registered cases between January 2000 and April 2019 (among ~ 7 million population). Incidences per union (smallest administrative unit) were calculated using geospatial population density estimates. A geospatial Poisson model was used to detect incidence hotspots over three (overlapping) 10-year timeframes: 2000–2009, 2005–2014 and 2010–2019. Ordinal regression models were used to assess whether patient characteristics were significantly different for cases outside hotspots, as compared to cases within weak (i.e., relative risk (RR) of one to two), medium (i.e., RR of two to three), and strong (i.e., RR higher than three) hotspots.

**Results:**

New case detection rates dropped from 44/100 000 in 2000 to 10/100 000 in 2019. Statistically significant hotspots were identified during all timeframes and were often located at areas with high population densities. The RR for leprosy was up to 12 times higher for inhabitants of hotspots than for people living outside hotspots. Within strong hotspots (1930 cases among less than 1% of the population), significantly more child cases (i.e., below 15 years of age) were detected, indicating recent transmission. Cases in hotspots were not significantly more likely to be detected actively.

**Conclusions:**

Leprosy showed a heterogeneous distribution with clear hotspots in northwest Bangladesh throughout a 20-year period of decreasing incidence. Findings confirm that leprosy hotspots represent areas of higher transmission activity and are not solely the result of active case finding strategies.
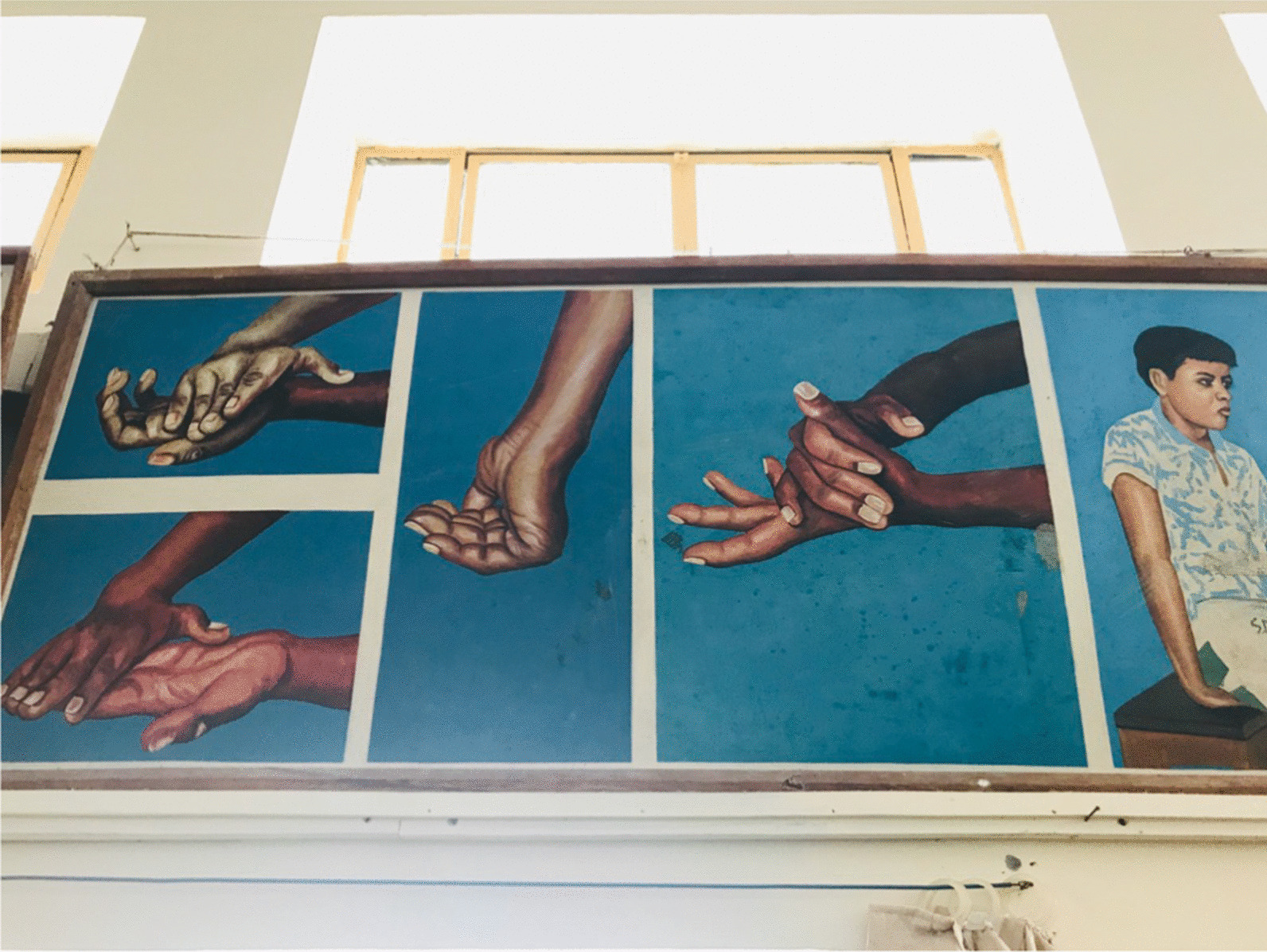

**Supplementary Information:**

The online version contains supplementary material available at 10.1186/s40249-021-00817-4.

## Background

Leprosy, or Hansen’s disease, is a chronic neglected tropical disease (NTD) caused by *Mycobacterium leprae*. This often-stigmatizing disease can lead to deformity and disability when left untreated. Leprosy can occur among people of all ages and primarily affects the skin, peripheral nerves, mucosa of the upper airway, and the eyes [1]. Main symptoms include ﻿red or hypopigmented skin patches with thickened nerves, definite loss of sensation, muscle weakness and numbness in hands and feet (disability grade 1) and, in a later stage, deformity of hands and feet and vision loss (disability grade 2) [1, 2]. Leprosy can also be present ﻿with no anaesthesia and no visible deformity or damage (grade 0) [2]. Clinical manifestation is usually enough for prompt diagnosis of leprosy. Patients with one to five skin patches are classified as paucibacillary (PB), and those with more than five patches, or with a positive slit-skin smear bacterial load, as multibacillary (MB) [3]. The spread takes place via respiratory droplets and requires close contact [4]. The disease is mildly contagious, MB more than PB leprosy, with an average incubation period of four years in PB and eight years in MB patients. However, it can take as long as 20 years for symptoms to develop [3, 5].

Fortunately, early treatment with multidrug therapy (MDT) is associated with good curability of leprosy and can halt progression of disability [6, 7]. Since the utilization of MDT for leprosy treatment in the late 1980s, the disease has been targeted for elimination [7, 8]. Global newly detected cases of leprosy decreased from over five million cases in the mid-1980s to about 200 thousand in 2018 [9, 10]. Most new cases can be found in India, Brazil, Indonesia, Bangladesh, and Ethiopia [11, 12]. Within countries, leprosy usually shows a heterogeneous distribution [13–15]. Generally, communities from resource-limited settings are most severely affected by the disease [16]. Bangladesh is high-endemic for leprosy since many decades, with known foci in the southeast (Cox’s Bazar), central (around Dhaka capital) and northwest (around Rangpur, Nilphamari, and Saidpur city). In 2018 and 2019, around four thousand new cases were detected annually [17].

Albeit considerable progress in leprosy control over the years, transmission of *M. leprae* appears to continue unabated, indicated by the static new case detection rate over the past 15 years globally. Innovative scientific methods and operational approaches are needed to both better understand the underlying geospatial epidemiological features of leprosy and the mechanisms of transmission, and to develop effective preventive interventions such as chemo- and immunoprophylaxis [18]. Targeting areas or foci with a high leprosy endemicity, also indicated as ‘hotspots’, is hoped to further increase the efficacy of such interventions. Although previous research has shown that substantial geospatial heterogeneity exists in leprosy occurrence within many endemic regions [19–21], it is yet unclear what factors exacerbate the regional leprosy burden and how the observed geospatial heterogeneity affects patient characteristics. We hypothesize that hotspots of high leprosy incidence within endemic areas to some extent represent foci of active transmission of the disease. Hence, we expect more child cases, i.e., below 15 years, in these foci as an indicator for recent transmission, since cases in this age group are known to have been recently infected.

The aim of this study is to identify and characterize leprosy hotspots in northwest Bangladesh over the past 20 years and explore whether hotspots represent foci of active leprosy transmission, by comparing case characteristics by endemicity levels, to gain further insight into the epidemiology of leprosy.

## Methods

### Data

The leprosy data come from four districts in northwest Bangladesh—Nilphamari, Panchagarh, Rangpur and Thakurgoan—an area of 7200 km^2^ and population of over 7 million [22]. The study population consists of all leprosy cases diagnosed and registered for leprosy treatment between 1 January 2000 and 30 April 2019. Leprosy cases are registered through the Rural Health Programme (RHP) of The Leprosy Mission International Bangladesh (TLMIB), located in Nilphamari; a referral centre specialized in the detection and treatment of leprosy in co-operation with the government leprosy control programme. Leprosy cases were identified through both passive (voluntary reporting through one of the 24 local health clinics and referral) and active detection (door-to-door surveys and screening of case contacts). All new cases were confirmed by a medical officer and the standard MDT combination was given according to the national guidelines. Up to 20 household, neighbour and close social contacts were screened during biennial follow-up visits [23–25]. Leprosy-positive contacts (based on clinical manifestation and/or positive skin smear results) were registered to the programme. It is estimated that roughly 95% of individuals living in this region who were treated for leprosy over the past 35 years, are known to the RHP.

For each leprosy case, main disease characteristics were recorded, as well as the age, sex and place of residence (i.e., administrative areas: district, upazila and union name). The GPS coordinates of the house of leprosy cases at time of diagnosis were collected retrospectively, between October 2018 and May 2019. This was done by 40 field staff employees, who had a 3-day training course. Coordinates were collected through the app MapIt Pro (https://mapitgis.com/) on smartphones and were automatically sent to a shared database. GPS coordinates of the health clinics and major cities were collected online, with Google Maps (https://www.google.com/maps).

**Fig. 1:**
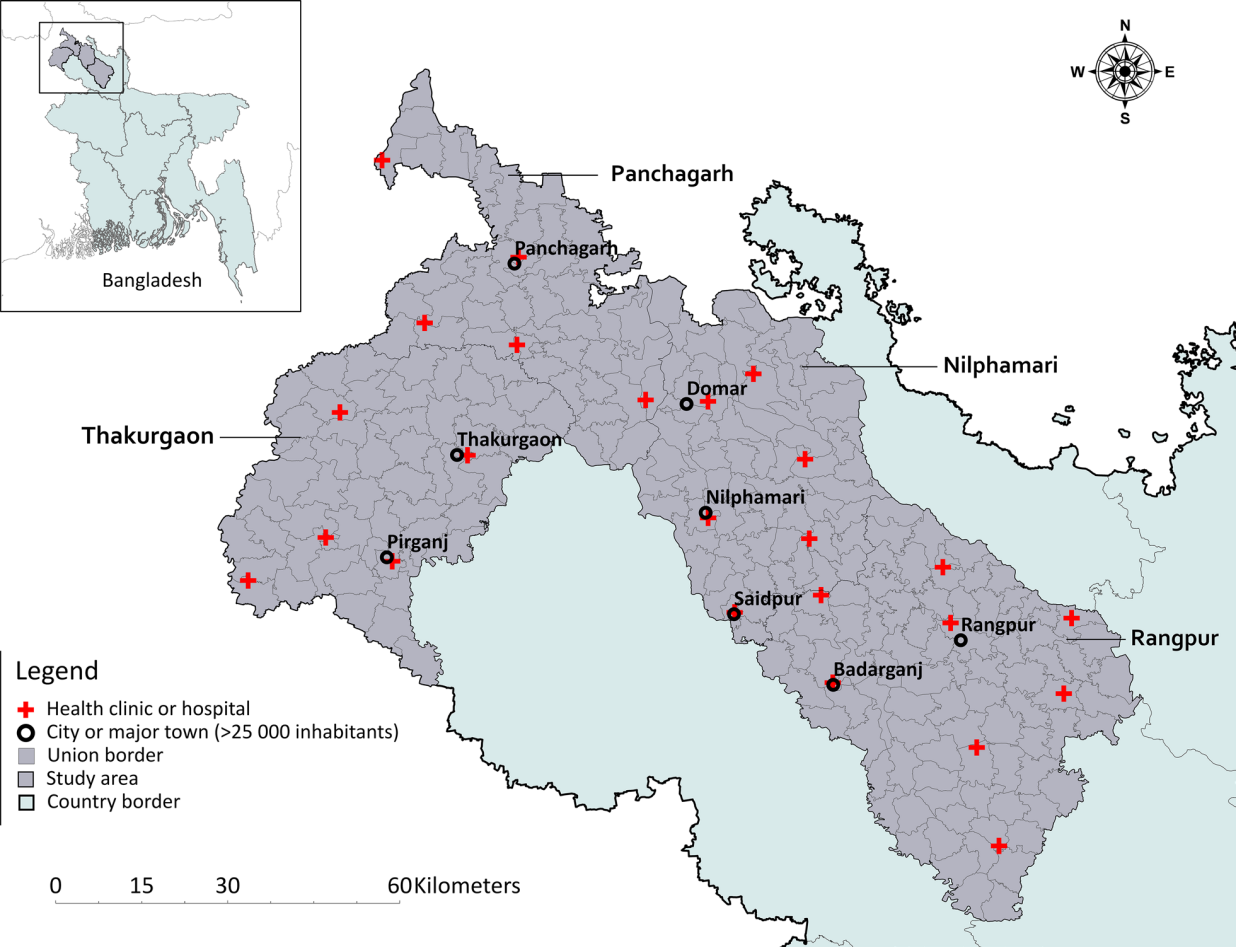
Overview of the study area in northwest Bangladesh, which covers four districts, Nilphamari (64 unions), Panchagarh (45 unions), Rangpur (86 unions), and Thakurgoan (54 unions)

The region is mainly rural, with six big towns or cities and multiple rivers that cross the landscape. Bangladesh has a sub-tropical monsoon climate, characterized by wide seasonal fluctuations in rainfall, humidity and temperatures. An overview of the study area is provided in Fig. 1.

Population data were available from WorldPop (www.worldpop.org), where the number of population per 100 m^2^ grid square was estimated for 2005, 2010, 2015 and 2020, with national totals adjusted to match UN population division estimates (http://esa.un.org/wpp/), census 2011 [26]. Annual population growth rates per district were obtained from the Bangladesh Bureau of Statistics population data.

### Statistical analyses

Three (overlapping) 10-year timeframes were selected: timeframe one (2000–2009), timeframe two (2005–2014) and timeframe three (2010–2019). Incidence rates per 100 000 capita were calculated at the union-level, the smallest official administrative area, with an average area surface of around 30 km^2^. The number of cases per union was acquired using registered administrative area names. If available, GPS coordinates were used for verification (proportion of cases with GPS coordinates available per year are shown in Additional file 1: Figure S1). Union-level population sizes were acquired for each timeframe by extrapolating WorldPop population estimates to the other years of the study. We estimated the annual population per union for each year of the study, by aggregating 100 m^2^ population estimates and extrapolating to other years of the study, using district-level annual growth rates, details are provided in Additional file 1: Figures S2 and S3.

Hotspots were identified based on union-level incidence. Incidence levels were chosen instead of case counts, to correct for heterogeneity in the underlying population at risk. We searched for purely spatial hotspots within the three separate 10-year timeframes. The relatively long time window of 10 years with temporal overlap was selected, because of the long incubation period of leprosy [3, 5] and often unclear time of disease onset. Empirical Bayesian smoothing was applied to take away possible uncertainty in the incidence estimates, due to smaller population numbers [27]. A Poisson model was used through spatial scan statistics to detect significant (*P*-value < 0.05) hotspots, defined in this study as foci with a significantly higher number of leprosy cases relative to the underlying population at risk (as compared to an equal distribution of cases throughout the area, relative to the underlying population) [28]. The model provides a series of Poisson-based draws against which leprosy incidence rates are compared. Under the null hypothesis, stating that cases are randomly dispersed throughout the study area, the expected number of cases is proportional to the population size of the study area. The probability that a hotspot did not originate by chance was determined by Monte Carlo hypothesis testing based upon the most likely hotspot, a likelihood ratio test was used to obtain a *P*-value for the most likely hotspot. We searched for hotspots that covered a maximum of 20% of the area and did not allow for geographical overlap of hotspots.

We compared the demographic, disease and location characteristics of newly detected cases located outside hotspots, and within significant hotspots: divided into hotspots with a relative risk (RR) of one to two (weak hotspots), two to three (medium hotspots), or three or higher (strong hotspots). A case was defined as falling into a hotspot, when being in a hotspot during at least one timeframe corresponding with the year of detection of the case. We used univariate and multivariate ordinal logistic regression models, in which hotspot ranked categories were included as dependant variable. The following independent variables were included: sex, age, type of leprosy, skin smear result, disability grade at time of diagnosis, Euclidian (straight-line) distance to nearest leprosy health facility, Euclidian distance to nearest major city or town, and 100 m^2^ population size around the case. Age was transformed to a categorical variable (below 15, 15–24, 25–34, 35–44, 45–54, and 55 and older). We used more specific age categories than the WHO standard age groups—distinguishing between child cases (below 15 years of age) and adult cases (15 years or older)—to allow for observation of possible heterogeneities in age distribution of cases by leprosy burden. Univariate models were adjusted for year of detection of the case. The multivariate model was adjusted for mode of detection and year of detection of the case. The union was added as a random effect in all models to capture unexplained incidence heterogeneity at this level.

Data entry was done in Microsoft Access. Empirical Bayesian Smoothing of the case and population data was applied in GeoDa (https://geodacenter.github.io/) version 1.12 (Anselin, Santa Barbara, CA, USA). Hotspot analyses were performed in SaTSan (https://www.satscan.org/) version 9.6 (Kulldorff, Boston, MA, USA). R version 3.4.3 (RStudio, PBC, Boston, MA, USA) was utilized for general data processing and statistical analyses. Processing and visualisation of the spatial data was done in ArcGIS Pro version 2.1.0 (ESRI, Redlands, CA, USA).

## Results

### Data characteristics

From January 2000 to April 2019, a total of 20 623 cases were diagnosed with leprosy in the study area in northwest Bangladesh. Most cases were diagnosed in Nilphamari and Rangpur districts (38.4% and 37.9% respectively), where roughly 25% and 42% of the population lives respectively. Less than half (44.5%) of all cases were female. At time of diagnosis, 2589 cases (12.6%) were below the age of 15. Overall, 74.3% of cases were diagnosed with PB leprosy. Of the 25.7% cases with MB leprosy, 38.3% had a positive skin smear result at time of diagnosis (9.9% of all cases). Overall, the majority of cases were found through passive reporting, either through voluntary registration (75.6%) or referral (10.2%). Another 14.1% of cases were detected through surveys or contact screening. An overview of the case characteristics is provided in Table 1.

**Table 1:** Demographic, disease and location characteristics of leprosy cases in northwest Bangladesh, detected from January 2000 to April 2019

	All cases
Total	*N* = 20 623 (100%)
Sex	
Male	11 649 (56.5%)
Female	8974 (44.5%)
Age at diagnosis (years)	
Below 15	2589 (12.6%)
15 to 24	4035 (19.6%)
25 to 34	4027 (19.2%)
35 to 44	3916 (19.0%)
45 to 54	3334 (16.2%)
55 and older	2722 (13.2%)
Group	
Paucibacillary (PB)	15 319 (74.3%)
Multibacillary (MB)	5301 (25.7%)
Unknown	3
Skin smear	
Negative	16 546 (89.1%)
Positive	2029 (9.9%)
Unknown	2048
Disability	
Grade 0	17 809 (86.4%)
Grade 1	1441 (7.0%)
Grade 2	1367 (6.6%)
Unknown	6
Mode of detection	
Survey	1524 (7.4%)
Referred	2107 (10.2%)
Voluntary	15 582 (75.6%)
Contact	1390 (6.7%)
Unknown	20
District	
Nilphamari	7928 (38.4%)
Panchagarh	2001 (9.7%)
Rangpur	7825 (37.9%)
Thakurgaon	2869 (13.9%)

GPS coordinates were collected retrospectively for 53.6% (*n* = 11 044) of the cases present in the database. Compared to the full database, less GPS coordinates were collected for cases located in Nilphamari (31.5% versus 38.4%) and more in Rangpur (47.5% versus 37.9%). All other characteristics were comparable to the full database (see Additional file 1: Table S2).

### Overall epidemiology and leprosy hotspots

**Fig. 2:**
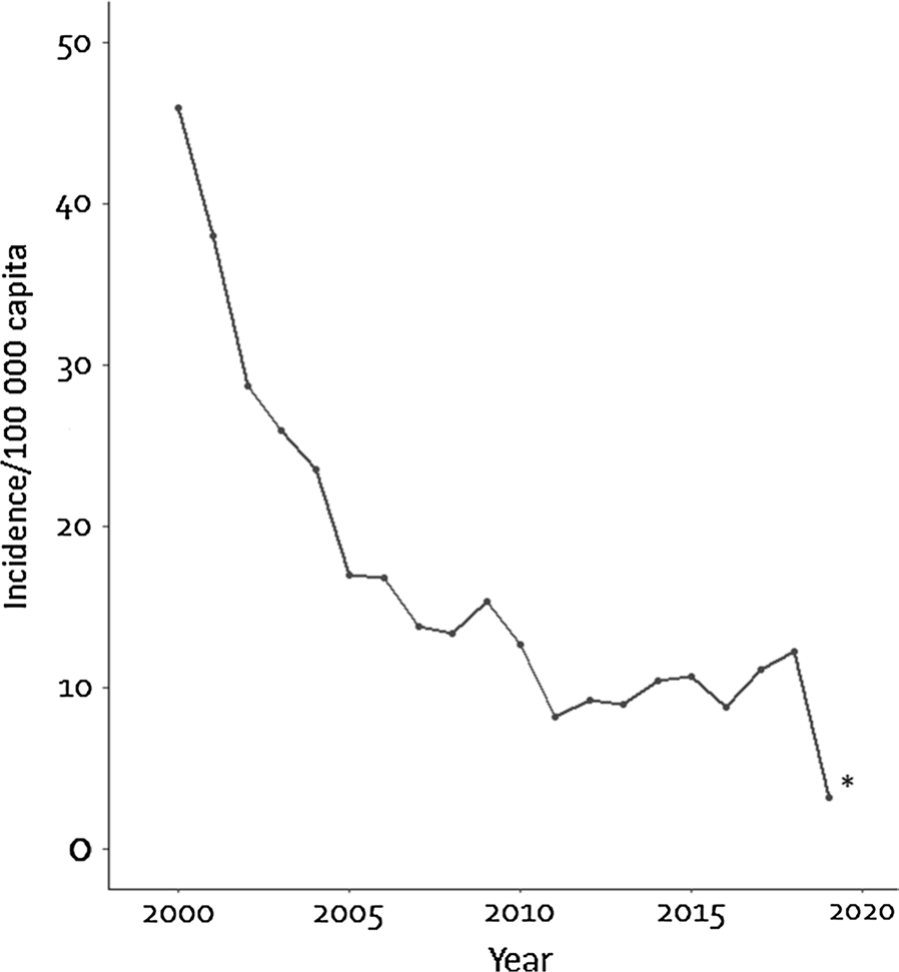
Trends over time of leprosy incidence. The 2019 data include cases that were registered over a four-month timeframe (from 1 January 2019 to 30 April 2019) and were extrapolated (multiplied by three) to represent the estimated number of cases for the whole year of 2019 (*)

Figure 2 shows that leprosy incidence was highest in the first year of the study and steeply declined during the following ten years; from 44 cases/100 000 to 16 cases/100 000 capita. During the second half of the study period, January 2010 to April, 2019, incidence levels fluctuated between eight and 12 cases/100 000 capita. The mean annual leprosy incidence was 17 cases/100 000 capita.

**Fig. 3:**
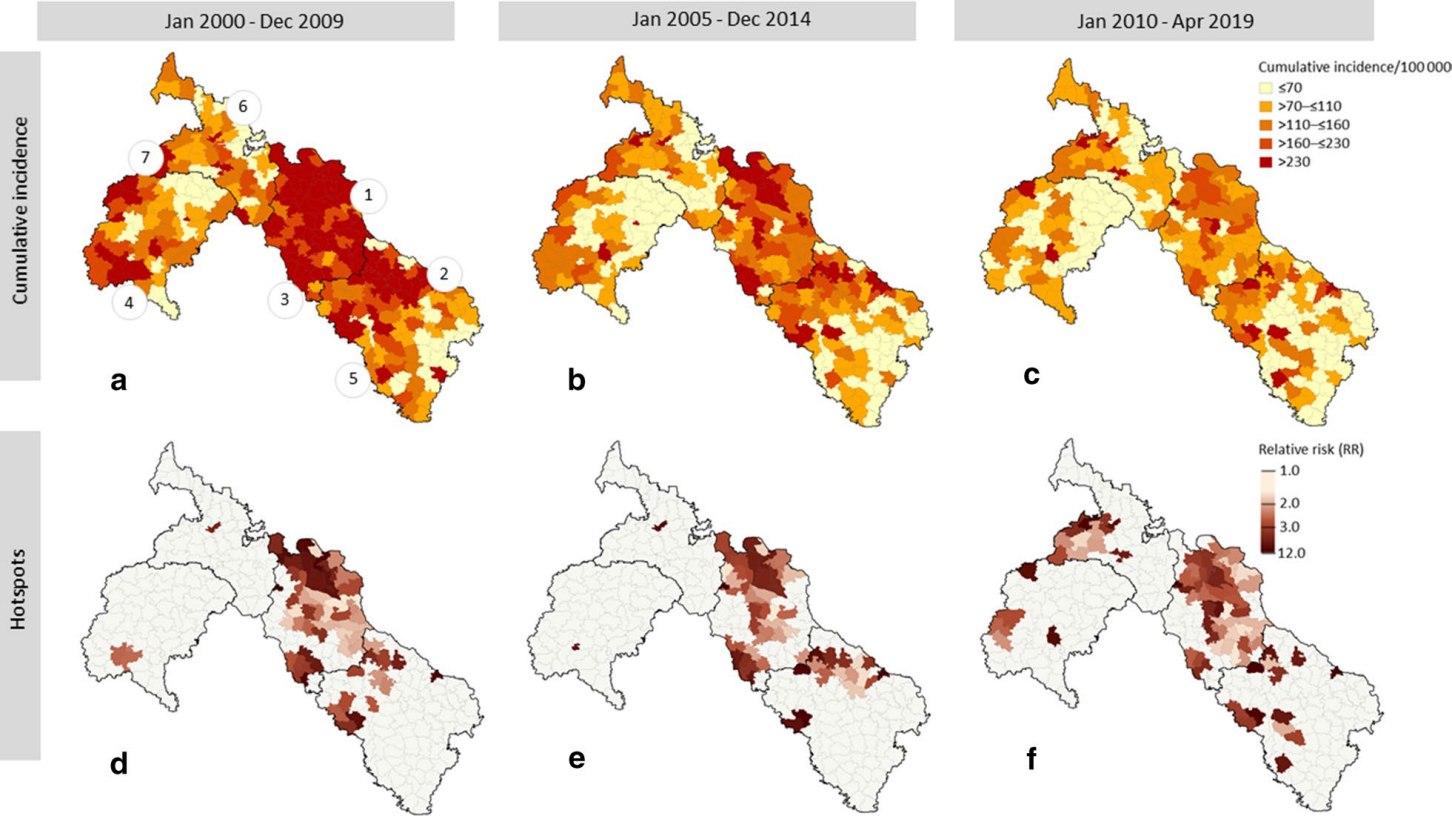
Cumulative (smoothed) incidence levels (panel **a**, **b** and **c**) and hotspots detected with spatial scan statistics (panel **d**, **e** and **f**) in northwest Bangladesh between January 2000 and April 2019 for three overlapping 10-year timeframes

The decrease in overall incidence is also reflected in the union-level incidence rates (Fig. 3, panel a). In timeframe one (2000–2009), cumulative incidence was highest (more than 230/100 000 capita) in the north of Nilphamari district (location 1), around Rangpur (location 2) and around Nilphamari and Saidpur (location 3). About a third (34%) of unions fell into this highest incidence category during timeframe one, 10% during timeframe two and only 4% during timeframe three. Overall, cumulative incidence seemed highest around major cities and health clinics (locations 1 to 7). Generally, areas of low incidence remained low throughout the different timeframes.

Despite the lower incidence in more recent years of the study, significant hotspots remained present throughout all three timeframes (Fig. 3, panel b). Hotspots (i.e. significantly more leprosy cases relative to the underlying population at risk, as compared to an equal or random distribution of cases relative to the underlying population) were mostly located in the north of Nilphamari district (location 1), around the cities Rangpur (location 2), around Nilphamari and Saidpur (location 3) and around Pirganj in Thakurgaon district (location 4) and at the southwest border of Rangpur district (location 5), where also health clinics are located. A small hotspot was present around Panchagarh city (location 6). New hotspots pop up in timeframe three at the western border with India, both in Panchagarh and Thakurgoan district (location 7). Here, incidence remained high, whereas incidence levels in the rest of the area were lower in timeframe three, compared to the earlier timeframes.

Out of the 20 623 cases reported throughout the study period, 9734 were located outside of hotspots 10 369 cases were located within a hotspot at time of detection. For 520 cases (2.5%) the exact location was unknown. Although hotspots on average capture 22% of the total area (1561 km^2^) and 24% of the population (around 1.7 million), 52% of the cases were inside a hotspot at the time of detection. The relative risk of having leprosy was up to twelve times higher for inhabitants of hotspots, compared to those living outside hotspots. Hotspots captured one to 20 (out of 249) unions and covered surfaces of 6 up to 546 km^2^. Maps of the union-level population size, cumulative case counts, unsmoothed cumulative incidence levels and a detailed overview of hotspot characteristics are provided in Additional file 1: Figure S4 and Additional file 1: Table S3.

Of the 10 369 cases detected within significant hotspots, 5324 cases (25.8% of all cases) were detected within hotspots with a RR of two to three and 1930 of these cases (9.4% of all cases) were located in hotspots with a RR of three or higher. Whereas all significant hotspots capture about 24% of the population in the area, hotspots with a RR of two or higher captured about 8% of the total population, and the strongest hotspots (RR of 3 or higher) captured less than 1% of the total population.

### Leprosy case characteristics

**Fig. 4:**
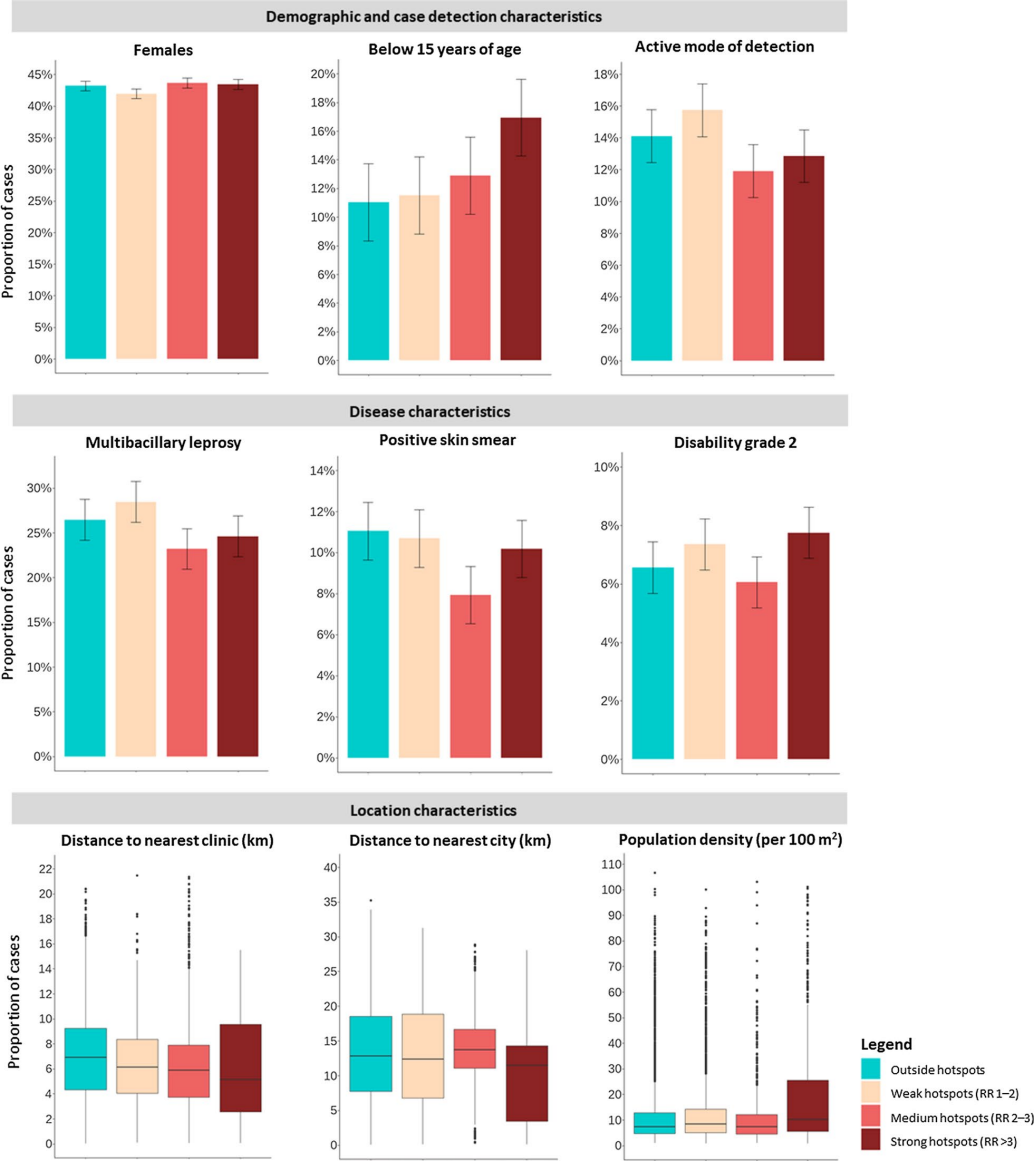
Demographic, disease and location characteristics of leprosy cases outside of hotspots and within weak, medium or strong hotspots and in northwest Bangladesh

Demographic, disease, and location characteristics of leprosy cases outside of hotspots and within weak, medium and strong hotspots are presented in Fig. 4, univariate and multivariate ordinal regression results are presented in Table 2, and the raw numbers and percentages are provided in Additional file 1: Table S4. A clear upward trend was observed in the age distribution of cases: in strong hotspots, cases were significantly more often below 15 years of age as compared to cases in less strong hotspots and outside of hotspots (from 11.5% outside of hotspots to 17.6% in strong hotspots, *P* < 0.001). The mode of detection, leprosy group (PB or MB), skin smear result, and disability grade were only modestly different for cases outside hotspots as compared to cases within strong hotspots. In strong hotspots, cases significantly less often had a positive skin smear results, as compared to cases detected outside of hotspots—remaining significant in the multivariate model (10.2% versus 11.1%, *P* = 0.011). For cases within strong hotspots, the median Euclidean proximity to the nearest health clinic was significantly lower, compared to cases living outside of hotspots (6.0 km [standard deviation (SD) = 3.9 km] versus 6.9 km [SD = 3.4 km], *P* < 0.001). Cases within strong hotspots were, overall, detected significantly closer to the nearest city (9.9 km [SD = 5.8 km] versus 13.4 km [SD 7.3 km], *P* < 0.001) and population density in strong hotspots was significantly higher (18.7 [SD = 9.8] versus 11.7 [SD = 6.2] inhabitants per 100 m^2^, *P* < 0.001). Although significant in the univariate model, the adjusted multivariate model shows that cases within strong hotspots were not significantly more likely to be detected actively, as compared to cases outside of hotspots (14.1% versus 12.8%, *P* = 0.700).

**Table 2:** Outcomes of univariate and multivariate ordinal logistic regression models of the association between demographic, disease and location characteristics of leprosy cases for different endemicity levels

	Univariate models		Multivariate models	
Covariate	a*OR*^a^ (95% *CI*)	*P*-value	a*OR*^b^ (95% *CI*)	*P*-value
Sex				
Male	1		1	
Female	1.00 (0.95–1.06)	0.882	0.95 (0.88–1.03)	0.245
Age at diagnosis (years)				
Below 15	1.25 (1.15–1.35)	< 0.001***	1.26 (1.12–1.43)	< 0.001***
15 and older	1		1	
Group				
Paucibacillary (PB)	1		1	
Multibacillary (MB)	0.92 (0.87–0.98)	0.006**	0.92 (0.83–1.02)	0.071
Skin smear				
Negative	1		1	
Positive	0.91 (0.82–1.02)	0.100	0.83 (0.73–0.96)	0.011*
Disability				
Grade 0	1		1	
Grade 1	1.06 (0.96–1.17)	0.262	1.04 (0.89–1.21)	0.497
Grade 2	1.04 (0.94–1.16)	0.432	1.05 (0.90–1.22)	0.353
Proximity to nearest clinic (km)	1.24 (1.13–1.34)	< 0.001***	1.26 (1.16–1.37)	< 0.001***
Proximity nearest city (km)	0.97 (0.97–0.98)	< 0.001***	0.98 (0.98–0.99)	< 0.001***
Population size (per 100 m^2^)	1.01 (1.00–1.01)	< 0.001***	1.00 (1.00–1.01)	0.004**
Mode of detection				
Active	1.13 (1.06–1.23)	< 0.001***	0.98 (0.87–1.10)	0.700
Passive	1		1	

We compared cases located outside of hotspots with cases within weak (RR lower than two), medium (RR of two to three) or strong (RR of three or higher) hotspots

a*OR* adjusted odds ratio, *CI* confidence interval

Significance codes: 0 ‘***’ 0.001 ‘**’ 0.01 ‘*’ 0.05 ‘.’ 0.1 ‘’ 1

^a^Models were adjusted for year of detection of the case (fixed effect) and union of residence (random effect)

^b^Models were adjusted for mode of detection, year of detection of the case (fixed effects) and union of residence (random effect)

## Discussion

Our results show that, despite the overall decrease in leprosy incidence over the past two decades in northwest Bangladesh, spatial heterogeneity in incidence remains present. Significant high-risk foci or ‘hotspots’ were detected during all three 10-year (overlapping) timeframes and mostly stay in the same areas: i.e., predominantly at areas closer to cities and with higher population density. Within strong hotspots, there were significantly more child cases, and the patients lived significantly closer to the nearest health clinic. Other demographic and disease characteristics of cases were not considerably different for strong hotspots.

This study is unique in that it represents a longitudinal dataset over a two-decade timespan from one of the most densely populated leprosy endemic rural areas in the world. The available data allowed for in-depth data analysis to expand the existing knowledge on the spatial epidemiology of leprosy, in particular comparison of leprosy characteristics to further explore how hotspots might relate to active transmission of leprosy. Within strong hotspots, there were significantly more cases below the age of 15 years, indicating that these areas have active ongoing recent transmission [29].

The shorter distance to clinics in hotspots may explain the higher detection rate in these areas, due to a combination of increased access to awareness programmes, often organized at school and communities in the more densely populated areas and around the clinics, and increased access to leprosy care. Active case detection activities in leprosy are known to increase the number of cases in an area substantially, as it would detect patients that otherwise would remain undetected for years. Our findings did not show a significant difference in the mode of detection (active versus passive case detection) for hotspots, thus the identified hotspots are not solely the result of the active case detection.

Two other mechanisms that put people at higher risk of acquiring leprosy could potentially explain the higher rates of active ongoing transmission within hotspots. Leprosy is a disease of poverty [30], and weakened immune systems due to absence of clean drinking water, proper sanitation and hygiene measures (WASH) and lack of healthy nutrition have long since been recognized as important determinants of leprosy [31, 32]. Frequent floods in the region, as a result of the sub-tropical monsoon climate with periods of heavy rainfall, might affect WASH and access to food in some parts of the study area. Moreover, ethnic variation and migration flows was found to cause heterogeneity in leprosy susceptibility in India [33] and Brazil [34]. After the Bangladesh war for independence in the early 1970s, a large refugee camp was created near Saidpur city [35], where ‘stateless’ Bihari refugees still live up-to-date. The northwest Bangladesh region also knows large seasonal migration flows: the 1 in 3 poor households are estimated to migrate every year during the monsoons to cope with seasonal deprivation [36]. It remains unknown if, and to what extent, these mechanisms influence the observed geospatial epidemiology of leprosy in the study area.

New case detection rates and incidence trends reported in the area follow global leprosy trends, showing a steeper decrease in the years after 2000 that stabilizes five to ten years later and continues in a more or less stable fluctuating trend [37, 38]. This trend, as in many other leprosy endemic countries, likely reflects the result of a period of often intensified active case finding activities starting in the 1980s and 1990s to register all leprosy cases before the year 2000, for which the leprosy elimination target was set [7, 39]. Intensified control activities continued after 2000 to register and treat the remaining hidden cases, and the new case detection rate likely became more clearly a representation of the actual incidence rate of leprosy some years after 2000. The gradual increase in the number of reported cases after 2012 and 2013, might be due to the roll out of extra case finding surveys, intended to reach the case inclusion targets for the intervention studies that started in those years [24]. The heterogeneous spatial epidemiological patterns of leprosy incidence that are shown in this study, are also known to be present in India [20, 40, 41] and Brazil [42–48]. The high rate of PB cases is well-known for this area [49] and is thought to be driven by genetic differences relative to Brazil and Southeast Asia, for example, where MB cases predominate [50].

Geospatial methods are proven to be important for targeting active case finding strategies and public health interventions at hotspot areas, as this strategy could yield a high proportion of new cases [8]. In these areas, innovations could be implemented to halt transmission and address underlying inequalities, such as early detection of cases by contract-tracing [5] and single-dose rifampicin prophylaxis for close contacts of leprosy cases [24, 51], but also conditional cash transfers [52] and local campaigns to increase knowledge and awareness about leprosy and reduce leprosy-related stigma [53]. Although the current definition of ‘hotspots’ and accompanied geospatial methodologies are valuable as precision public health tools, finding areas where leprosy is significantly spatially clustered does not seem sufficient to identify underlying drivers of leprosy transmission. In our dataset with many new cases and a large underlying population at risk, modestly higher incidence levels often lead to a significantly higher relative risk, already being identified as hotspots in the applied analyses. Under this assumption, hotspots proved to be not distinct enough from the rest of the area to explore them as drivers of leprosy transmission. Therefore, we further subdivided the hotspots to identify the areas with the highest leprosy incidence, in this study indicated as ‘strong’ hotspots. This allowed us to point out ‘strong’ hotspots as areas of active leprosy transmission, a finding we otherwise would have missed. This highlights the need for critical assessment of the available hotspot detection methods to be able to get further insight into the epidemiology and transmission dynamics of leprosy, and possibly also for other NTDs.

Our study has several limitations. First, population estimates were calculated based on national and district-level growth rates. Therefore, we did not account for possible small-scale fluctuations, triggered by urbanization and other types of migration flows. However, the population is known to have been growing at a relatively stable rate throughout the past two decades throughout the whole area [22]. Second, due to the lack of precise demographic data for the underlying population at risk, we cannot exclude that the identified differences in age patterns among cases by hotspot category reflect similar differences in the age distribution of the underlying population. According to the latest population census (2011), about 32% of the population in Bangladesh is below 15 years of age [22]. Bangladesh is a low-income country with rapid economic development, which often leads to “urban drift”: young people who migrate to cities after their secondary school to seek employment. Although this leads to a lower average age in cities, larger proportions of school-aged children likely remain present in rural areas—whereas we identified larger proportions of child leprosy cases in and around urban areas. Third, not all leprosy cases were traced back to acquire GPS coordinates for this study. GPS coordinates could be collected for only over half of all the registered cases in the area and for relatively more cases living in Nilphamari, the district where the main clinic is located—potentially leading to some bias in geospatial analyses. Therefore, we identified hotspots based on the union locations, that were available for 97% of the cases, and used the GPS coordinates as verification. The distance to the nearest clinic and nearest city were acquired using GPS data if available, and were otherwise calculated using the coordinates of the centre of the union the case lived in. Although the first method is more precise, we do not expect significantly different results in our analyses, since the likelihood of having GPS coordinates available for cases living within hotspots was similar compared to for cases living outside of hotspots. Finally, throughout the study period, cases and case contacts have been recruited into various research trials and observational studies, of which COLEP, COCOA, and MALTALEP have been the largest [23–25, 49, 54]. The trials focused on chemoprophylaxis and/or immunoprophylaxis in contacts of leprosy index cases. Although some of the interventions were considered effective to reduce the transmission of *M. leprae* among contacts of leprosy cases [24], we do not expect a significant influence on the overall spatial epidemiological patterns observed in this study. Most cases were included in the COCOA study, which was observational. In total 3700 cases were part of the intervention or control arm of one of the intervention trials, and these cases are spread out over the study area and registered over a long time period.

Future research could focus on enhancing further improvement of case finding strategies and leprosy treatment to further optimize control of leprosy. Data on diagnosis-to-treatment time, number of health clinic visits, treatment completion, and treatment success rates, among other factors, could be analysed to further optimize existing leprosy programmes. More specifically, the association between delayed case finding or treatment delays and the existence of hotspots could be investigated. Furthermore, studies could focus on carrying out more in-depth hotspot location predictions, using open-source environmental and socioeconomic data, for example, to be able to target case finding strategies and other interventions effectively. This would especially be of interest for extrapolation to areas with no or incomplete leprosy data, but good availability of (open-source) data on potential environmental predictors of leprosy hotspots. This approach has been widely used for other infectious diseases.

## Conclusions

Our findings suggest that, regard-less of the seemingly stable epidemiology of leprosy in the area up to date, strong hotspots remain present in northwest Bangladesh. The identified hotspots at this scale are, at least partially, representations of areas with higher transmission activity, as cases detected there are significantly more often below the age of 15 years, indicating recent transmission. We could verify that hotspots are not solely the result of active case finding strategies. Although moving in the right direction, continued efforts are needed to halt transmission of leprosy in high endemic areas, such as northwest Bangladesh, in order to reach the ultimate goal of leprosy elimination.

## Supplementary Information

**Additional file 1: Figure S1. **Trends over time of the number of new leprosy cases registered, where indicated whether or not GPS coordinates could be collected. **Figure S2. **Estimated annual population size per district.** Figure S3. **Estimated population density per union per 5-year time frame. **Table S2. **Demographic, disease and location characteristics of leprosy cases overall, and cases for which GPS coordinate were collected retrospectively in northwest Bangladesh, detected between January 2000 and April 2019. **Figure S4. **Mean population size (panels A, B, C), cumulative case counts (panels D, E, F) and unsmoothed cumulative incidence levels (panels G, H, I) and hotspots detected with spatial scan statistics with identification numbers (panels J, K, L) in northwest Bangladesh between January 2000 and April 2019. **Table S3. **Leprosy hotspots in northwest Bangladesh between January 2000 and April 2019, detected with spatial scan statistics (https://www.satscan.org/). The location identification numbers of hotspots are shown in Figure S3 (panels J to L). The area locations are shown in Figure 3. **Table S4**. Demographic, disease and location characteristics of leprosy cases in northwest Bangladesh, detected from January 2000 to April 2019.

## Data Availability

Data can be made available upon motivated request.

## Abbreviations

GPS: Global positioning system
MB: Multibacillary
MDT: Multidrug therapy
NTD: Neglected tropical disease
PB: Paucibacillary
RHP: Rural Health Programme
RR: Relative risk
TLMIB: The Leprosy Mission International Bangladesh
