# Supplementary material for: Geospatial epidemiology of leprosy in northwest Bangladesh: a 20-year retrospective observational study
Source: Infect Dis Poverty. 2021 Mar 22;10:36. doi: 10.1186/s40249-021-00817-4 (PMC7986508; doi:10.1186/s40249-021-00817-4)
Supplement: Supplementary file 1 — Additional file 1: Figure S1. Trends over time of the number of new leprosy cases registered, where indicated whether or not GPS coordinates could be collected. Figure S2. Estimated annual population size per district. Figure S3. Estimated population density per union per 5-year time frame. Table S2. Demographic, disease and location characteristics of leprosy cases overall, and cases for which GPS coordinate were collected retrospectively in northwest Bangladesh, detected between January 2000 and April 2019. Figure S4. Mean population size (panels A, B, C), cumulative case counts (panels D, E, F) and unsmoothed cumulative incidence levels (panels G, H, I) and hotspots detected with spatial scan statistics with identification numbers (panels J, K, L) in northwest Bangladesh between January 2000 and April 2019. Table S3. Leprosy hotspots in northwest Bangladesh between January 2000 and April 2019, detected with spatial scan statistics (https://www.satscan.org/). The location identification numbers of hotspots are shown in Figure S3 (panels J to L). The area locations are shown in Figure 3. Table S4. Demographic, disease and location characteristics of leprosy cases in northwest Bangladesh, detected from January 2000 to April 2019. [file 40249_2021_817_MOESM1_ESM.docx]

**SUPPLEMENTARY MATERIAL**

**Supplementary Figure 1. Trends over time of the number of new leprosy cases registered, where indicated whether or not GPS coordinates could be collected.** The 2019 data include cases that were registered over a four-month timeframe (from 1 January 2019 to 30 April 2019) and were extrapolated (multiplied by three) to represent the estimated number of cases for the whole year of 2019 (light grey).


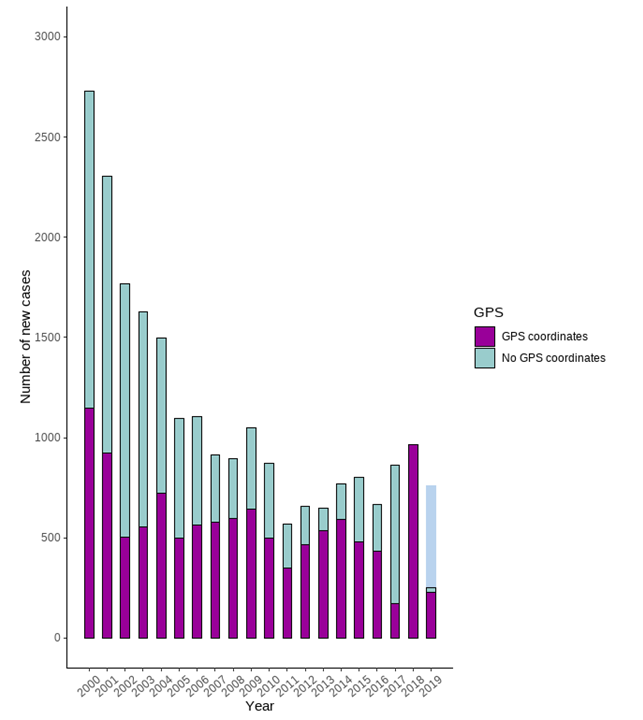


**Supplementary Figure 2. Estimated annual population size per district.** Annual population estimates per union were acquired by extrapolating the Census 2011 WorldPop 100 m^2^ grid square raster data ([www.worldpop.org](http://www.worldpop.org)). Annual population growth rates were calculated based on a weighted average of three available population data sources: UN 2001 to 2015 (<https://population.un.org/wpp/>, available at the national level); World Population Review 2000, 2005, 2010 and 2015 to 2019 (<http://worldpopulationreview.com/countries/bangladesh-population/>, available at the national level); and Bangladesh Bureau of Statistics 1991, 2001, 2011 and 2016 (<http://www.citypopulation.de/Bangladesh-Mun.html>, available at the district level). For the missing years, growth rates were calculated based on the available rates from other years from the same data source, where an exponentially growing population was assumed. For 2019, the population size of 2018 was used.

**
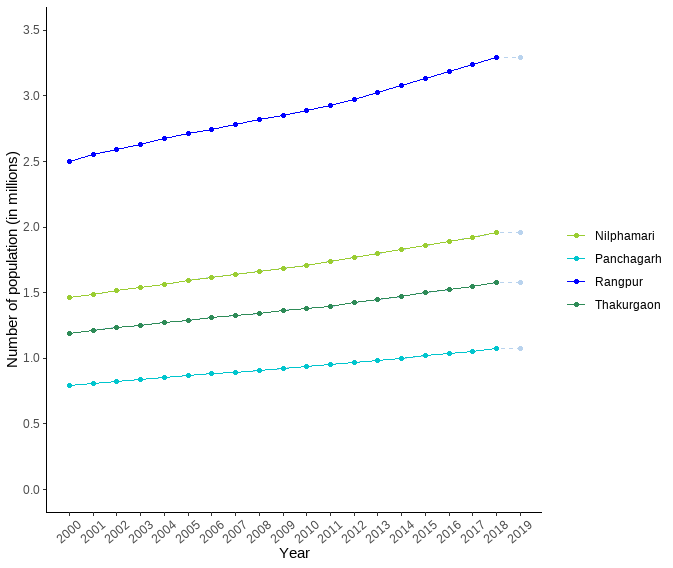
**

**
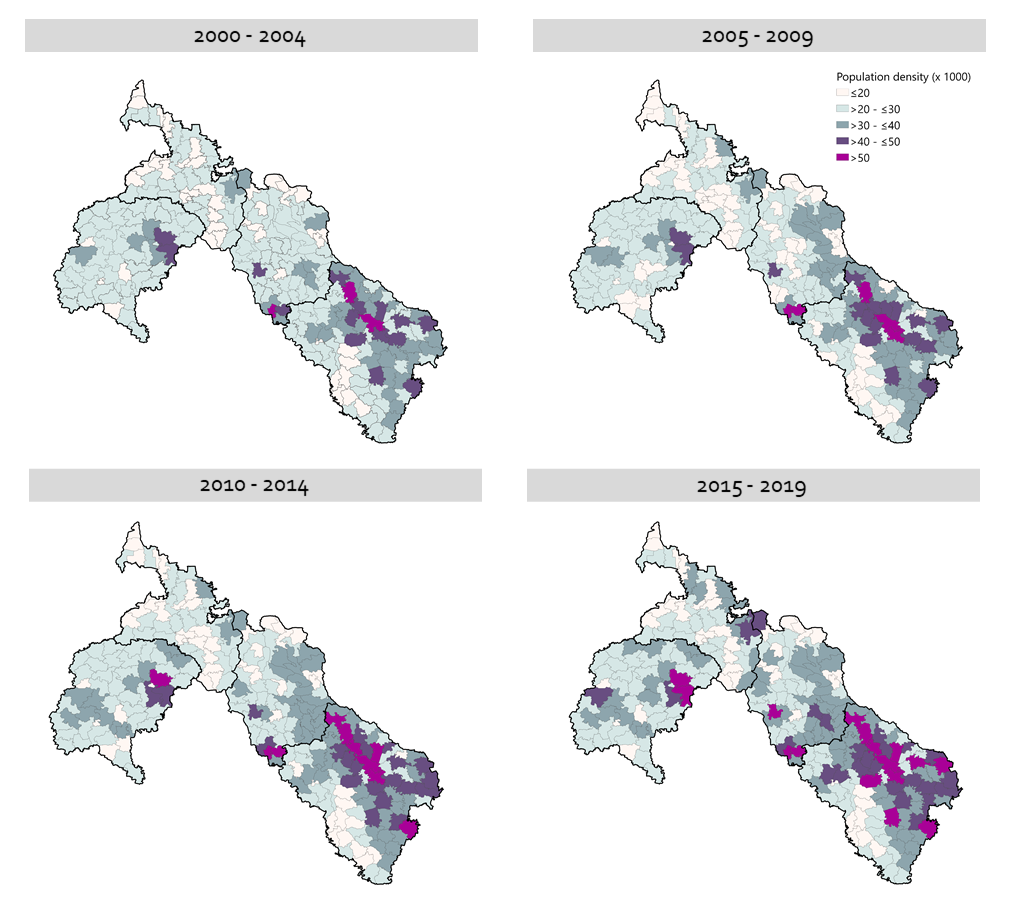
 Supplementary Figure 3. Estimated population density per union per 5-year time frame.**

**Supplementary Table 2. Demographic, disease and location characteristics of leprosy cases overall, and cases for which GPS coordinate were collected retrospectively in northwest Bangladesh, detected between January 2000 and April 2019.**

|  | **All registered cases** | **Cases with GPS coordinates available** |
| --- | --- | --- |
| **Total** | N = 20,623 (100%) | N = 11,044 (100%) |
| **Sex** |  |  |
| Male | 11,649 (56.5%) | 6,269 (56.8%) |
| Female | 8,974 (44.5%) | 4,775 (43.2%) |
| **Age at diagnosis (years)** |  |  |
| Below 15 | 2,589 (12.6%) | 1,229 (11.1%) |
| 15 to 24 | 4,035 (19.6%) | 2,109 (19.1%) |
| 25 to 34 | 4,027 (19.2%) | 2,200 (19.9%) |
| 35 to 44 | 3,916 (19.0%) | 2,146 (19.4%) |
| 45 to 54 | 3,334 (16.2%) | 1,868 (16.9%) |
| 55 and older | 2,722 (13.2%) | 1,492 (13.5%) |
| **Group** |  |  |
| Paucibacillary (PB) | 15,319 (74.3%) | 8,393 (76.0%) |
| Multibacillary (MB) | 5,301 (25.7%) | 2,650 (24.0%) |
| Unknown | 3 | 1 |
| **Skin smear** |  |  |
| Negative | 16,546 (89.1%) | 8,871 (89.7%) |
| Positive | 2,029 (9.9%) | 1,015 (10.3%) |
| Unknown | 2,048 | 1,158 |
| **Disability** |  |  |
| Grade 0 | 17,809 (86.4%) | 9,545 (86.4%) |
| Grade 1 | 1,441 (7.0%) | 724 (6.6%) |
| Grade 2 | 1,367 (6.6%) | 770 (7.0%) |
| Unknown | 6 | 5 |
| **Mode of detection** |  |  |
| Survey | 1,524 (7.4%) | 1,136 (10.3%) |
| Referred | 2,107 (10.2%) | 1,440 (13.1%) |
| Voluntary | 15,582 (75.6%) | 7,966 (72.2%) |
| Contact | 1,390 (6.7%) | 489 (4.4%) |
| Unknown | 20 | 13 |
| **District** |  |  |
| Nilphamari | 7,928 (38.4%) | 3477 (31.5%) |
| Panchagarh | 2,001 (9.7%) | 906 (8.1%) |
| Rangpur | 7,825 (37.9%) | 5,247 (47.5%) |
| Thakurgaon | 2,869 (13.9%) | 1,414 (12.8%) |

**Supplementary Figure 4.** **Mean population size (panels A, B, C), cumulative case counts (panels D, E, F) and unsmoothed cumulative incidence levels (panels G, H, I) and hotspots detected with spatial scan statistics with identification numbers (panels J, K, L) in northwest Bangladesh between January 2000 and April 2019.**

**
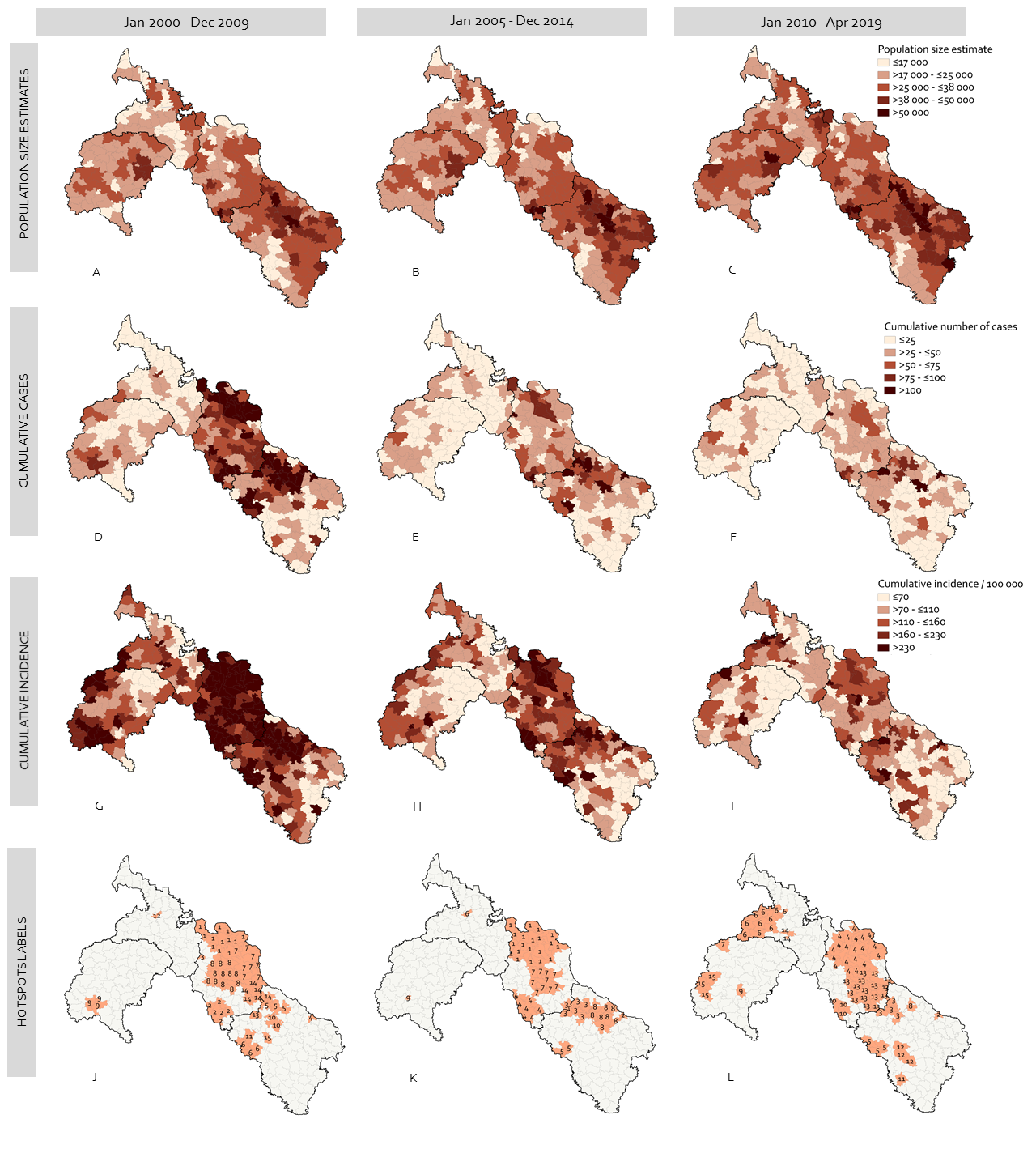
**

**Supplementary Table 3. Leprosy hotspots in northwest Bangladesh between January 2000 and April 2019, detected with spatial scan statistics (**[**https://www.satscan.org/**](https://www.satscan.org/)**).** The location identification numbers of hotspots are shown in **Supplementary Figure 3** (panels J to L). The area locations are shown in **Figure 3**.

| **Timeframe** | **Identification number** | **Location (as in Figure 3)** | **Number of unions** | **Hotspot surface (km^2^)** | **Population** | **Observed number of cases** | **Expected number of cases** | **Relative risk (RR)** | **Log likelihood** | **P-value** |
| --- | --- | --- | --- | --- | --- | --- | --- | --- | --- | --- |
| **2000-2009** |  |  |  |  |  |  |  |  |  |  |
|  | 1 | 1 | 12 | 296.04 | 233,938 | 1,215 | 489 | 2.63 | 400.80 | <0.001 |
|  | 2 | 3 | 6 | 178.48 | 176,397 | 987 | 368 | 2.81 | 368.58 | <0.001 |
|  | 3 | 1 | 1 | 30.86 | 6,777 | 172 | 14 | 12.29 | 272.53 | <0.001 |
|  | 4 | 2 | 1 | 30.03 | 21,353 | 202 | 45 | 4.58 | 148.55 | <0.001 |
|  | 5 | 2 | 3 | 58.54 | 120,169 | 557 | 251 | 2.27 | 141.37 | <0.001 |
|  | 6 | 5 | 3 | 93.52 | 68,486 | 333 | 143 | 2.36 | 92.65 | <0.001 |
|  | 7 | 1 | 8 | 245.68 | 194,698 | 697 | 407 | 1.75 | 88.27 | <0.001 |
|  | 8 | 1 | 12 | 337.99 | 252,519 | 783 | 309 | 1.51 | 56.17 | <0.001 |
|  | 9 | 4 | 3 | 96.24 | 53,361 | 194 | 112 | 1.75 | 25.17 | <0.001 |
|  | 10 | 2 | 2 | 48.33 | 82,366 | 268 | 172 | 1.57 | 23.11 | <0.001 |
|  | 11 | 5 | 1 | 28.63 | 33,933 | 135 | 71 | 1.91 | 22.97 | <0.001 |
|  | 12 | 6 | 1 | 22.11 | 14,839 | 75 | 31 | 2.43 | 22.31 | <0.001 |
|  | 13 | 2 | 1 | 20.63 | 25,617 | 107 | 54 | 2.01 | 20.73 | <0.001 |
|  | 14 | 1 | 6 | 98.53 | 199,154 | 533 | 416 | 1.29 | 15.54 | <0.001 |
|  | 15 | 5 | 1 | 22.53 | 21,369 | 82 | 45 | 1.84 | 12.53 | <0.001 |
| **Total** | **-** | **-** | **61** | **1,608** | **1,504,976** | **6,340** | **2 927** | **-** | **-** | **-** |
| **2005-2014** |  |  |  |  |  |  |  |  |  |  |
|  | 1 | 1 | 20 | 447.39 | 45,1138 | 979 | 546 | 1.90 | 150.85 | <0.001 |
|  | 2 | 2 | 1 | 14.26 | 23,043 | 158 | 28 | 5.75 | 144.93 | <0.001 |
|  | 3 | 2 | 6 | 166.89 | 22,6151 | 562 | 273 | 2.13 | 121.22 | <0.001 |
|  | 4 | 3 | 8 | 216.58 | 21,9677 | 518 | 266 | 2.01 | 97.31 | <0.001 |
|  | 5 | 5 | 2 | 61.90 | 44,110 | 181 | 53 | 3.44 | 94.35 | <0.001 |
|  | 6 | 6 | 1 | 28.17 | 16,194 | 63 | 20 | 3.23 | 30.27 | <0.001 |
|  | 7 | 1 | 10 | 278.48 | 254,815 | 416 | 308 | 1.37 | 17.62 | <0.001 |
|  | 8 | 2 | 8 | 207.69 | 466,036 | 690 | 564 | 1.24 | 14.13 | <0.001 |
|  | 9 | 4 | 1 | 23.29 | 11,848 | 36 | 14 | 2.52 | 11.50 | 0.002 |
| **Total** | **-** | **-** | **57** | **1,444.65** | **1,713,012** | **3,603** | **2 072** | **-** | **-** | **-** |
| **2010-2019** |  |  |  |  |  |  |  |  |  |  |
|  | 1 | 1 | 1 | 6.99 | 7,973 | 87 | 7 | 12.10 | 136.63 | <0.001 |
|  | 2 | 2 | 1 | 11.79 | 24,824 | 120 | 23 | 5.37 | 103.42 | <0.001 |
|  | 3 | 2 | 4 | 98.22 | 143,460 | 290 | 131 | 2.27 | 73.48 | <0.001 |
|  | 4 | 1 | 20 | 570.47 | 514,023 | 741 | 469 | 1.65 | 72.83 | <0.001 |
|  | 5 | 5 | 3 | 90.53 | 79,620 | 160 | 73 | 2.23 | 39.54 | <0.001 |
|  | 6 | 7 | 10 | 301.22 | 212,354 | 309 | 194 | 1.62 | 29.98 | <0.001 |
|  | 7 | 7 | 1 | 37.42 | 23,626 | 63 | 22 | 2.94 | 26.24 | <0.001 |
|  | 8 | 2 | 1 | 25.80 | 42,355 | 89 | 39 | 2.32 | 24.07 | <0.001 |
|  | 9 | 4 | 1 | 27.30 | 17,841 | 51 | 16 | 3.15 | 23.60 | <0.001 |
|  | 10 | 3 | 3 | 71.42 | 85,537 | 140 | 78 | 1.81 | 20.13 | <0.001 |
|  | 11 | 5 | 1 | 29.99 | 19,101 | 42 | 17 | 2.42 | 12.41 | 0.001 |
|  | 12 | 5 | 1 | 96.24 | 59,263 | 94 | 54 | 1.75 | 12.17 | 0.001 |
|  | 13 | 1 | 17 | 379.53 | 499,555 | 557 | 456 | 1.24 | 11.28 | 0.003 |
|  | 14 | 6 | 1 | 19.65 | 15,203 | 32 | 14 | 2.31 | 8.64 | 0.028 |
|  | 15 | 7 | 3 | 125.35 | 97,658 | 130 | 89 | 1.47 | 8.33 | 0.040 |
| **Total** | **-** | **-** | **68** | **1,891.92** | **1,842,393** | **2,905** | **1 682** | **-** | **-** | **-** |

**Supplementary Table 4. Demographic, disease and location characteristics of leprosy cases in northwest Bangladesh, detected from January 2000 to April 2019.**

|  | **Outside of hotspots** | **Weak hotspots**  **(relative risk 1-2)** | **Medium hotspots**  **(relative risk 2-3)** | **Strong hotspots**  **(relative risk >3)** |
| --- | --- | --- | --- | --- |
| **Total** | N = 9,734 (100%) | N = 5,045 (100%) | N = 3,394 (100%) | N = 1,930 (100%) |
| **Sex** |  |  |  |  |
| Male | 5,424 (56.3%) | 2,871 (57.5%) | 1,901 (56.0%) | 1,075 (55.7%) |
| Female | 4,216 (43.7%) | 2,118 (42.5%) | 1,493 (44.0%) | 855 (44.3%) |
| **Age at diagnosis (years)** |  |  |  |  |
| Below 15 | 1,119 (11.5%) | 609 (12.1%) | 450 (13.3%) | 339 (17.6%) |
| 15 to 24 | 1,977 (20.3%) | 930 (18.4%) | 634 (18.7%) | 403 (20.9%) |
| 25 to 34 | 1,945 (20.0%) | 980 (19.4%) | 681 (20.1%) | 332 (17.2%) |
| 35 to 44 | 1,855 (19.1%) | 978 19.4%) | 643 (18.9%) | 342 (17.7%) |
| 45 to 54 | 1,540 (15.8%) | 853 (16.9%) | 578 (17.0%) | 272 (14.1%) |
| 55 and older | 1,298 (13.3%) | 695 (13.8%) | 408 (12.0%) | 232 (12.6%) |
| **Group** |  |  |  |  |
| Paucibacillary (PB) | 7,192 (73.9%) | 3,671 (72.8%) | 2,620 (77.2%) | 1,474 (76.4%) |
| Multibacillary (MB) | 2,539 (26.1%) | 1,374 (27.2%) | 774 (22.8%) | 456 (23.6%) |
| Unknown | 3 | 0 | 0 | 0 |
| **Skin smear** |  |  |  |  |
| Negative | 7,629 (88.9%) | 4,107 (89.3%) | 2,917 (92.0%) | 1,581 (89.8%) |
| Positive | 948 (11.1%) | 492 (10.7%) | 252 (8.0%) | 179 (10.2%) |
| Unknown | 1,157 | 446 | 225 | 170 |
| **Disability** |  |  |  |  |
| Grade 0 | 8,460 (86.9%) | 4,299 (85.2%) | 2,945 (86.8%) | 1,675 (86.8%) |
| Grade 1 | 655 (6.7%) | 373 (7.4%) | 237 (7.0%) | 137 (7.1%) |
| Grade 2 | 617 (6.3%) | 370 (7.4%) | 211 (6.2%) | 118 (6.1%) |
| Unknown | 2 | 3 | 1 | 0 |
| **Mode of detection** |  |  |  |  |
| Survey | 715 (7.4%) | 439 (8.7%) | 196 (5.8%) | 138 (7.2%) |
| Referred | 1,090 (11.2%) | 539 (10.7%) | 268 (7.9%) | 138 (7.2%) |
| Voluntary | 7,301 (75.1) | 3,670 (72.8%) | 2,698 (79.6%) | 1,538 (79.8%) |
| Contact | 621 (6.4%) | 391 (7.8%) | 227 (6.7%) | 114 (5.9%) |
| Unknown | 7 | 6 | 5 | 2 |
| **District** |  |  |  |  |
| Nilphamari | 1,532 (15.7%) | 2,917 (57.8%) | 2,218 (65.4%) | 1,023 (53.0%) |
| Panchagarh | 1,534 (15.8%) | 232 (4.6%) | 90 (2.7%) | 115 (6.0%) |
| Rangpur | 4,256 (43.7%) | 1,604 (31.8%) | 1,019 (30.0%) | 737 (38.2%) |
| Thakurgaon | 2,412 (24.8%) | 292 (5.8%) | 67 (2.0%) | 55 (2.8%) |
| **Distance nearest clinic** (mean [SD]) | 6.9 (3.4) | 6.2 (3.0) | 6.2 (3.6) | 6.0 (3.9) |
| **Distance nearest city** (mean [SD]) | 13.4 (7.3) | 12.8 6.9) | 13.6 (4.5) | 9.9 (5.8) |
| **Population density per 100 m^2^** (mean [SD]) | 11.7 (6.2) | 13.0 (7.1) | 10.1 (5.0) | 18.7 (9.8) |
